# Identification and Compensation of Dynamic Interaction in a Non-Contact Dual-Stage Actuator System

**DOI:** 10.3390/s19051053

**Published:** 2019-03-01

**Authors:** Shaokai Wang, Jinxin Hu, Changqi Li, Jiubin Tan

**Affiliations:** 1 Centre of Ultra-precision Optoelectronic Instrument Engineering, Harbin Institute of Technology, Harbin 150080, China; 18s001011@stu.hit.edu.cn (J.H.); 18B901029@stu.hit.edu.cn (C.L.); jbtan@hit.edu.cn (J.T.); 2 Key Lab of Ultra-precision Intelligent Instrumentation (Harbin Institute of Technology), Ministry of Industry and Information Technology, Harbin 150080, China

**Keywords:** dual-stage actuator system, dynamic interaction, force feedforward, position-dependent disturbance forces, velocity-dependent disturbance forces

## Abstract

Dynamic interaction seriously limits the overall performance of a Dual-Stage Actuator (DSA) system. This paper aims to identify and compensate for the dynamic interaction in a non-contact DSA system. The effects of the interaction in the non-contact DSA system are initially classified as non-contact position-dependent disturbance forces (PDDFs) and velocity-dependent disturbance forces (VDDFs). The PDDFs in the three degrees of freedom (DoFs) motion space between the two stages of the DSA system are directly identified in the time domain, and VDDFs are indirectly identified in the form of damping values in frequency domains. The feedforward networks of the force are subsequently applied to compensate the PDDFs and VDDFs, which are indexed with relative displacement and velocity, respectively. Experiments are finally conducted to investigate the effectiveness of compensation, which infers that the final positioning error in the time domain can be reduced from 260 nm to 130 nm with PDDFs and VDDFs compensation. The gain of the interaction transfer is decreased in the frequency range of up to 45 Hz with PDDFs and VDDFs compensation. With this method, some weak dynamic interaction can be completely compensated for by the force feedforward compensation, and the positioning accuracy of non-contact DSA systems can be greatly improved.

## 1. Introduction

A Dual-Stage Actuator (DSA) system generally exists in the types of measuring equipment that simultaneously require both a long stroke and high precision. The DSA maintains a Long-Stroke (LS) (or Macro, Coarse, Primary) actuator stage and a Short-Stroke (SS) (or Micro, Fine, Secondary) actuator stage. These two redundant stages are typically connected in series. The LS actuator stage is expected to generate the large stroke and the SS is expected to generate the fast dynamic and high-resolution stroke. Therefore, an overall DSA system can output a fast response and high motion precision within a large stroke. Dual stage was first developed in the 1980s [1,2,3]. It has been widely applied in various fields, such as precision manufacturing [4,5,6,7], precision measurement [8], precision positioning [9,10], robotics [11], and data storage devices [12,13]. However, many experimental verifications [6,14,15] have inferred that the unnecessary and inevitable dynamic interaction between two stages can degrade the overall performance of the whole system. Thus, identification and compensation of the dynamic interaction are crucial for a DSA system.

For a contact DSA system, the positioning error of the LS is transferred by dynamic interaction between LS and SS, and results in a positioning error of the SS. The dynamic interaction is usually in the form of the force interaction, which includes the reaction force between the stages and the dynamic interaction force caused by dynamic model coupling of stages. Hodac et al. [16] designed a high compliance of the SS actuator stage’s suspension to reduce the impact of the reaction force acting on the LS actuator stage. In the DSA system proposed in Reference [17] for an optical disk system, a focus spring was designed to provide both high stiffness in the tracking direction and high compliance in the focus direction, which can suppress the interaction of the SS actuator stage’s inertia with the LS actuator stage’s motion accuracy. A similar generation method for high stiffness can also be found in Reference [18]. For the DSA system used in hard disks [19], a dual-input dual-output (DIDO) model is established based on a compensator and the loop shaping method is applied to compensate the interaction disturbance force. In References [20,21], the mechanical interaction is separated in the frequency domain, and the so-called PQ controller is adopted to reduce the DIDO system to two single-input/single-output (SISO) subsystems with the interaction compensation. An impact force controller was employed in Liu’s DSA system [22] to control the SS so as to overcome the interference behavior caused by the reaction force. Moreover, some novel flexure hinges [15,23,24] have also been designed based on a dynamic model in theory to reduce or eliminate the force interaction between the LS and the SS actuator stages. The DSA systems above contain physical contact connections between the LS and the SS actuator stages, which can therefore cause both the LS and SS actuator stages to be complex. 

A non-contact DSA system is therefore proposed, in which there is no mechanical connection between the LS and SS actuator stages. With the aid of the so-called ‘LS actuator stage follows SS actuator stage’ control strategy and the acceleration feedforward, the reaction force could be naturally compensated. Therefore, both the LS and the SS actuator stages could be respectively modeled as the SISO models with no force interaction. Furthermore, the positioning error of the LS actuator stage is approximately tens of micrometers, which is only one percent of the stroke of the SS actuator stage. Therefore, the non-contact DSA system, instead of the contact DSA system, can be used in applications requiring nanometer-scale tracking accuracy under large velocity conditions. However, some other weak interactions still exist, such as the leaked electromagnetic force, which seriously restrict the improvement of the non-contact DSA systems. Most of the existing research on non-contact DSA systems commonly ignores or simplifies the weak dynamic interaction between the LS and the SS actuator stages. In a DSA system [25] comprised of a linear motor as an LS actuator stage and a voice coil motor as an SS actuator stage, only the counter electromotive force of the voice coil between the LS and the SS actuator stages was discussed. Yonmook et al. [26] designed a reaction force compensator to compensate the multi-degrees of freedom (DoF) reaction force according to the actual relative position and velocity between the LS and the SS actuator stages. Additionally, in Reference [27], only the reaction force as force interaction is compensated for to prevent collisions. In these DSA systems, no other weak interactions were further analyzed, since they might not exist in their certain structures or may not critically influence their required performance. Many researchers have emphasized improving the LS motion performance [28,29,30,31] to suppress interactions in their DSA systems. However, in most DSA systems, it is complicated to further improve the optimization of the LS due to its naturally large mass, complex structure, and slow dynamics. 

In this paper, the specific mechanical structure and control strategy for a non-contact DSA system are firstly introduced. Additionally, the generation mechanism of the particular interaction is analyzed. Then, the effects of such interaction are classified into position-dependent disturbance forces (PDDFs) and velocity-dependent disturbance forces (VDDFs) for the first time, and identified in the time and the frequency domain, respectively. With the aid of a carefully designed classification methodology, both of the disturbance forces are compensated for by means of the force feedforward. Finally, the effectiveness of such compensation is evaluated experimentally. With this method, the influence of the weak dynamic interaction between the LS and the SS actuator stages can be compensated for by the force feedforward compensation without increasing the hardware cost of the system, and the positioning accuracy of non-contact DSA systems can be greatly improved. 

## 2. Description of the Dual-Stage Actuator (DSA) System

### 2.1. Mechanical Structure

**Figure 1:**
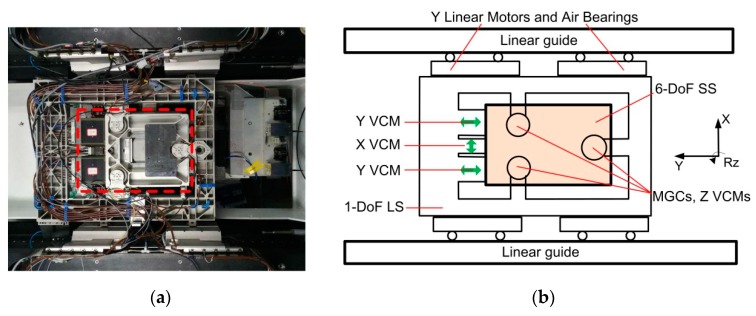
(**a**) Photograph and (**b**) schematic of the proposed non-contact Dual-Stage Actuator (DSA) system.

The construction of the dual-stage actuator is illustrated in Figure 1. The LS actuator stage is actuated by linear motors and guided by air-bearings in the Y direction, both of which are non-contact and friction-free. The SS actuator stage is a 6-DoFs magnetic levitation stage over a 1-DoF LS actuator stage with the aid of three Magnetic Gravity Compensators (MGCs). At the same time, each MGC is also integrated with one Voice Coil Motor (VCM) that can provide the Z-direction forces. The MGCs provide the magnetic force to approximately compensate for the gravity of the SS and maintain its levitation condition. Meanwhile, three VCMs of the MGCs corporately actuate the SS actuator stage to move in the Z, Rx, and Ry directions (vertical directions). Moreover, there are two other VCMs in the system that actuate the SS actuator stage in the Y direction, one VCM which actuates the stage in the X direction, and together the VCMs actuate the stage in the X, Y, and Rz directions (horizontal directions). All coil parts of the six VCMs are mounted on the LS actuator stage, and permanent magnet parts on the SS actuator stage. This mechanical structure prevents physical connection between the two stages, such as from electric cables and cooling hoses. In the Y direction, the SS and the LS actuator stages are complementary to achieve high motion precision within a large range. 

### 2.2. Measurement System and Control Strategy

The absolute position of the SS actuator stage is measured by means of interferometers in three horizontal directions, and by capacitive sensors in three vertical directions, while the absolute position of the LS actuator stage in the Y direction is measured by a linear encoder. The relative position between the LS and the SS actuator stages is measured by means of Hall sensors in three horizontal directions and eddy current sensors in three vertical directions. With the decoupling of the motion and the control force, the 6-DoF SS actuator stage is separated into six SISO systems independently. 

**Figure 2:**
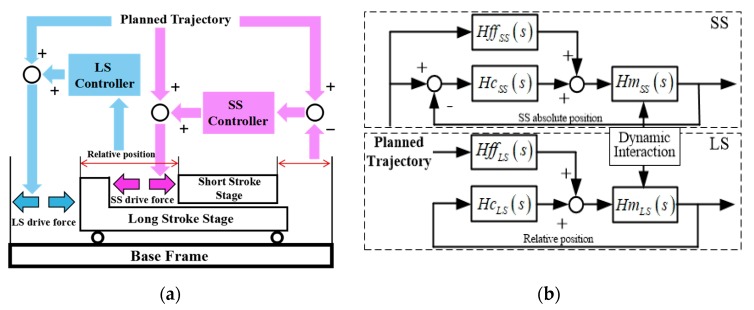
(**a**) Schematic and (**b**) control loops of control system in the Y direction.

The control strategy in the Y direction is shown in Figure 2. Since the dominant eigenfrequency of each stage is much higher than the corresponding control bandwidth, the mechanical plants could be properly described as *M^−1^s^−2^*. The planned trajectory of both the LS and the SS actuator stages is realized by acceleration feedforward *Hff*(*s*). To consider the reaction force of the SS actuator stage to the LS actuator stage, the LS actuator stage feedforward is defined as the total mass of the two stages, as *Hff_LS_*(*s*) *=* (*M_LS_ + M_SS_*)*s^2^*, while *Hff_SS_*(*s*) *= M_SS_s^2^* for the SS actuator stage. In the meantime, the SISO feedback controller *Hc*(*s*) is applied to compensate the on-line positioning error of each stage in each direction. The absolute position of the SS actuator stage and the relative position between the SS and the LS actuator stages are respectively taken as the feedbacks of the controllers of the SS and LS loops. There is another velocity loop in each SISO feedback controller, and the velocity is obtained by differentiating the displacement. Thus, the LS actuator stage is controlled to follow the SS actuator stage, just like the extended stroke of the SS actuator stage in the Y direction. In the other five directions, only the SS actuator stage moves in its limited stroke with similar control loops. Thus, all the dynamic interactions existing in the conventional DSA systems are naturally eliminated with the aid of the proposed non-contact mechanical structure. 

## 3. Generation Mechanism of Dynamic Interaction

**Figure 3:**
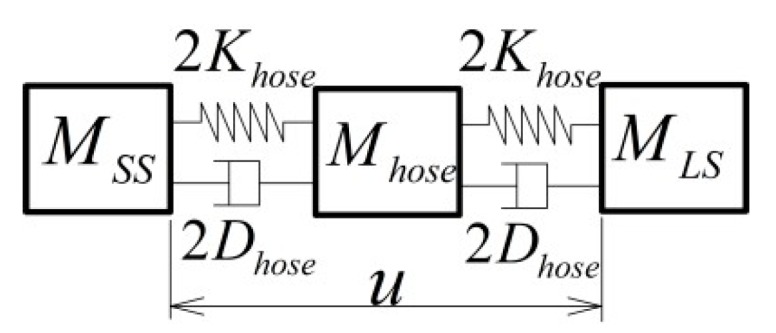
The mass–spring–damper model of vacuum hose.

Theoretically speaking, the output force of a VCM only depends on the input current; however, the output force is seriously affected by the physical connection, such as the presence of the vacuum hose, which is required by the vacuum clamped sample on the SS actuator stage. Although it is too compliant and light to influence the dynamics of the SS actuator stage, the disturbance force generated by the LS actuator stage can be transferred though the hose to the SS actuator stage. To analyze the interaction due to this vacuum hose, a simplified model of a mass–spring–damper is established, as shown in Figure 3.

When the hose mass accelerates with the LS and the SS actuator stages, the hose mass with the springs is pre-tensioned. When the moment of acceleration ends, the DSA system starts constant-velocity motion, and the pre-tension is released as vibration causing a disturbance force on the SS actuator stage. The disturbance force with settling time t can be subsequently calculated as $F_{hoseM}=M_{hose}ae^{-\xi \omega_{n}t}\sin\left(\omega_{d}t+\phi \right)$. If the settling time is long enough, this force can be neglected. However, the relative displacement u between the LS and the SS actuator stages in the constant-velocity scan still results in a disturbance force on the SS actuator stage through the stiffness *K_hose_* and the damping *D_hose_,* as $F_{hoseDK}=D_{hose}us+K_{hose}u$, which is position and velocity dependent.

**Figure 4:**
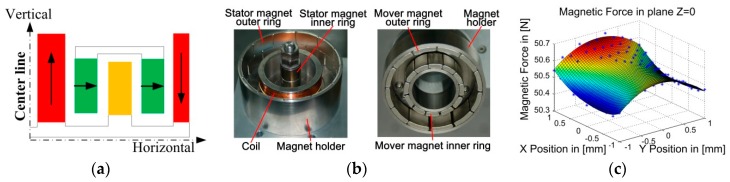
(**a**) Principle schematic, (**b**) pictures, and (**c**) tested magnetic levitation force of Magnetic Gravity Compensators (MGC) in the plane Z = 0.

The MGCs also lead to an unexpected interaction. The principle and structure of MGCs are illustrated in Figure 4a,b. Due to practical and theoretical errors [32], the magnetic fields of the MGCs are not uniform in both the horizontal and vertical directions. As a result, the magnetic levitation forces of MGCs cannot achieve absolute consistency. 

Taking one MGC as an example, the tested maglev force distributes in a horizontal plane, as shown in Figure 4c. Such inconsistency of the maglev force and parasitic horizontal force directly generates the PDDFs on the SS actuator stage due to the relative displacement between the LS and the SS actuator stages.

Moreover, the magnetic and electromagnetic interaction between the LS and the SS actuator stages result in interaction as well. Specifically, the permanent magnets of SS VCMs and MGCs interact with the ferromagnetic materials, and with non-ferromagnetic yet electrically conductive materials, of the LS actuator stage, which results in PDDFs and VDDFs with respect to the relative displacement and velocity, respectively. Additionally, the residual damping coefficient of the compensated back electromotive force of the SS actuator stage VCMs causes VDDFs. 

To summarize, all the interactions result from the PDDFs and VDDFs. To increase the positioning accuracy of the DSA system, it is therefore necessary to eliminate the PDDFs and VDDFs. According to the analyses of generation mechanism above, these disturbance forces are synthetically complicated in that they do not exist on any single component or any certain location of the SS actuator stage. Therefore, it is impossible to monitor and deal with each of them alone. However, these forces as a whole are able to be embodied as non-contact PDDFs and VDDFs in each direction with respect to the relative movement between the LS and the SS actuator stages. Some structural and performance parameters of the DSA system studied in this paper are shown in Table 1.

## 4. Identification and Compensation of Interaction

### 4.1. Identification of Disturbance Forces

#### 4.1.1. Position-Dependent Disturbance Forces (PDDFs)

**Figure 5:**
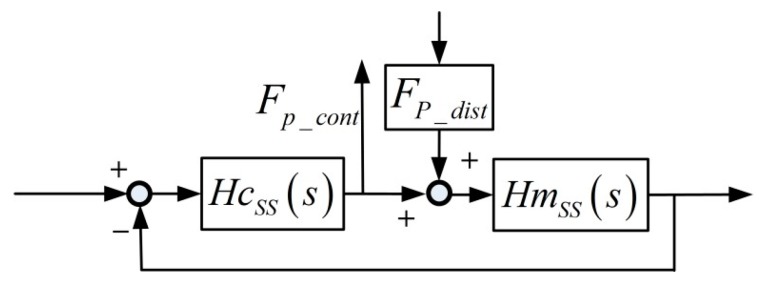
Relationship between position-dependent disturbance forces (PDDFs) and Short-Stroke (SS) controller forces.

To separate the PDDFs, they were experimentally identified in the time domain when the SS actuator stage moves at a very low and constant speed. Since only the relative speed affects VDDFs, it can be neglected under specially designed experimental conditions when the relative speed is small enough. The constant speed also implies that no extra acceleration force is generated. Thus, only PDDFs remain. The relationship between the PDDFs and the SS controller forces are illustrated in Figure 5.

**Figure 6:**
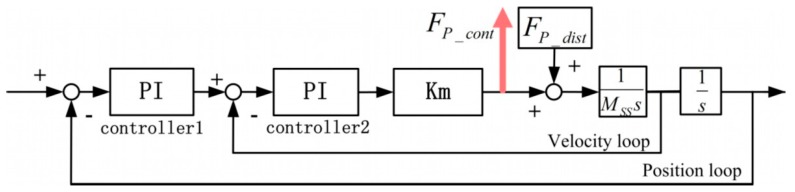
SS controller structure with the PDDFs as input and controller forces as output.

Specifically, the SS controller in each direction contains a velocity loop and a position loop, and each loop adopts a PI controller, as shown in Figure 6. 

Then, the PDDFs *F_p_dist_* can be calculated with the controller force *F_P_cont_* in the velocity loop, via Equation (1):

(1) $$\frac{F_{P\_cont}}{-F_{P\_dist}}=\frac{Km\left(Kp_{1}+\frac{Ki_{1}}{s}+s\right)\left(Kp_{2}+\frac{Ki_{2}}{s}\right)}{Km\left(Kp_{1}+\frac{Ki_{1}}{s}+s\right)\left(Kp_{2}+\frac{Ki_{2}}{s}\right)+M_{SS}s^{2}}$$

where *Kp* and *Ki* are PI controller parameters, *Km* is the equivalent motor constant in each direction, and M_SS_ is the mass or rotational inertia of mechanical plants.

According to the Bode diagram of the transfer of Equation (1) in each direction, the magnitude in the frequency range of up to 10 Hz almost equals 1. Therefore, when the frequency of the PDDFs is low enough at low speed, the controller forces equal the negative PDDFs.

**Figure 7:**
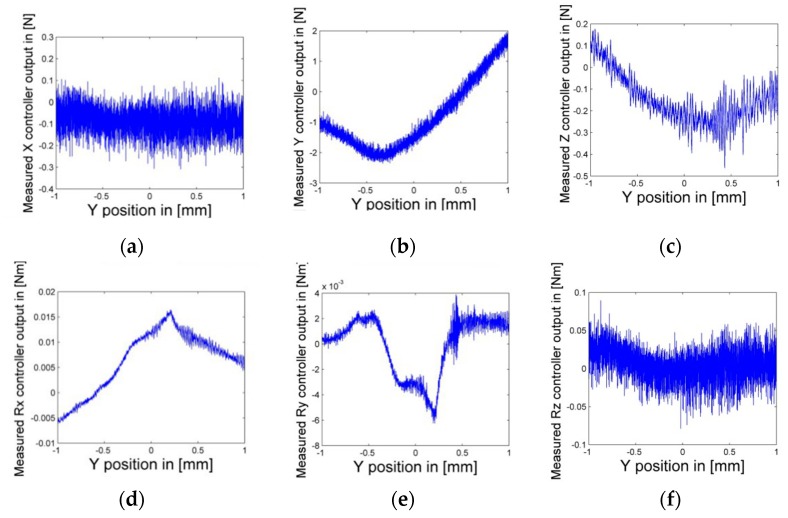
Recorded Short-Stroke (SS) controller forces: (**a**) X controller output, (**b**) Y controller output, (**c**) Z controller output, (**d**) Rx controller output, (**e**) Ry controller output, and (**f**) Rz controller output, with corresponding relative positions between the SS and the Long-Stroke (LS).

The LS is kept in a constant position and all its air bearings and linear motors are powered off to make sure no other external disturbance is introduced. The SS actuator stage moves fed with interferometers and the capacity sensors measure at a constant speed of 0.2 mm/s over the whole stroke in the Y direction. The SS controller forces in 6-DoFs are recorded corresponding to relative positions between the LS and the SS actuator stages, as measured by Hall and eddy current sensors. The one sampling period delay between them is compensated for in an off-line data storage process. For example, when the relative positions of the SS to the LS in the X and Z directions are X = −0.2 mm and Z = 0, the recorded results are as shown in Figure 7.

**Figure 8:**
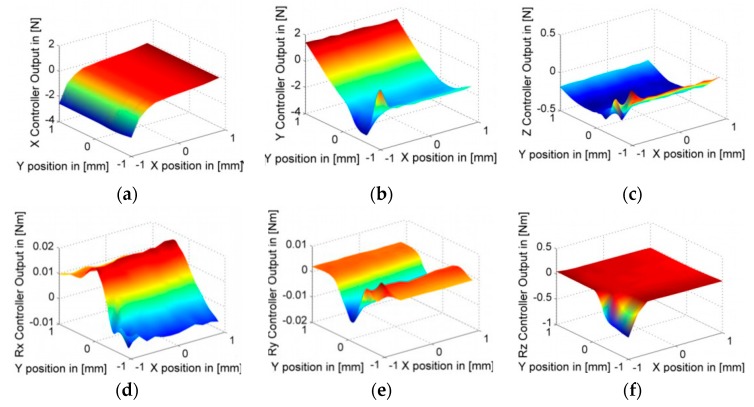
Distribution of PDDFs in (**a**) X, (**b**) Y, (**c**) Z, (**d**) Rx, (**e**) Ry, and (**f**) Rz degrees-of-freedom (DoF) in the horizontal plane for Z = −0.4 mm.

Figure 7 shows clearly that the PDDFs not only exist but also fluctuate greatly in some DoFs, such as 4N in the Y direction. The motion with the same parameters was repeated at different relative positions in the X and Z directions, specifically, X = ± 0.2 n (n = 0, 1, 2, 3, 4, and 5) mm and Z = −0.4, 0, and 0.4 mm. Thus, all these motions could cover the whole 3D motion space between the LS and the SS actuator stages (−1< X < 1, −1 < Y < 1, −0.4 < Z < 0.4 mm). To eliminate noise effects and ensure the reproducibility of the identification, controller forces corresponding to the relative position were measured 10 times at each relative position, and their mean values were calculated. The final results were integrated in three horizontal planes as Z = −0.4, 0, and 0.4 mm. As an example, Z = −0.4 is shown in Figure 8. Thus, the spatially discrete distribution of PDDFs on the SS actuator stage was obtained.

As shown in Figure 8, the variation gradient of most PDDFs in the Y direction is much greater than that in the X and Z directions, except for the small volume around the edge of X = −1 mm and Y = −1 mm. For this reason, the discrete intervals of identification in the Y direction are much smaller than those in the X and Z directions. Thus, for any relative position, 6-DoFs PDDFs on the SS actuator stage can be easily calculated with a linear interpolation method. For the special volume, extra identification with smaller discrete intervals in the X and Z directions are conducted.

#### 4.1.2. Velocity-Dependent Disturbance Forces (VDDFs)

Identification methods in the time domain are not available for VDDFs. The VDDFs on the SS actuator stage were determined by the relative velocity at different relative positions. It is impossible to change the relative velocity by a large margin at some relative positions within such limited 3D motion space between the SS and LS actuator stages. From another perspective, damping is the physical parameter relating force and velocity. If the spatial distribution of damping values could be obtained, the VDDFs are then indirectly identified.

For a generalized 1-DoF mechanical system including a damper and a mass in series, the relationship between the input displacement *u* of the damper, and the output acceleration *a* of the mass is Dus=ma. If the damping value is constant, the magnitude of the transfer function D=ma/us will be a horizontal line reflected in its Bode diagram. 

**Figure 9:**
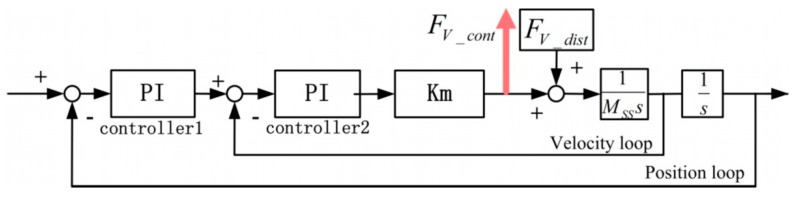
SS controller structure with the velocity-dependent disturbance forces (VDDFs) as input and controller forces as output.

When the PDDFs are compensated for, the only interaction disturbance forces left on the SS actuator stage are VDDFs. Then, it could be tested whether a certain damping value *D* exists at a certain relative position *u* between the LS and the SS actuator stages. The relationship between the VDDFs *F_V_dist_* acting on the SS actuator stage and controller force *F_V_cont_* is illustrated in Figure 9 and expressed as Equation (2):

(2) $$\frac{F_{V\_cont}}{F_{V\_dist}}=\frac{F_{V\_cont}}{uDs}=-\frac{Km\left(Kp_{1}+\frac{Ki_{1}}{s}+s\right)\left(Kp_{2}+\frac{Ki_{2}}{s}\right)}{Km\left(Kp_{1}+\frac{Ki_{1}}{s}+s\right)\left(Kp_{2}+\frac{Ki_{2}}{s}\right)+M_{SS}s^{2}}$$

Thus, as long as the relative displacement *u* contains enough information in the frequency domain, the damping value *D* can be obtained with the aid of the Bode diagram of transfer equation, as given in Equation (3), in which part *A* can be estimated with parameters of mechanical and control systems, and part *B* can be measured experimentally. The minus sign can be ignored when the absolute value of *D* is calculated.

(3) $$D=-\underset{A}{\underset{︸}{\frac{Km\left(Kp_{1}+\frac{Ki_{1}}{s}+s\right)\left(Kp_{2}+\frac{Ki_{2}}{s}\right)+M_{SS}s^{2}}{Km\left(Kp_{1}+\frac{Ki_{1}}{s}+s\right)\left(Kp_{2}+\frac{Ki_{2}}{s}\right)}}}\frac{1}{s}\underset{B}{\underset{︸}{\frac{F_{V\_cont}}{u}}}$$

In the discussed DSA system, the maximum acceleration of the SS actuator stage in the Y direction is 60 m/s^2^, which is much higher than the accelerations of 1 m/s^2^, 0.1 m/s^2^, 0.2 rad/s^2^, 0.2 rad/s^2^, and 1 rad/s^2^ in the X, Z, Rx, Ry, and Rz directions, respectively. Therefore, the relative velocity between the LS and the SS actuator stages in the Y direction is far higher than it is in other directions. Thus, only the VDDFs in the Y direction are required to be identified and can be neglected in other directions.

**Figure 10:**
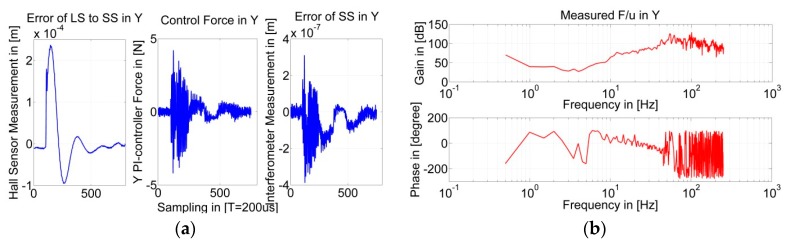
(**a**) Recorded relative position, SS controller force, and SS actuator stage positioning error; and (**b**) Bode diagram of the transfer function of controller force to relative position.

In the VDDF identification experiment, the LS actuator stage was positioned in the same location as in the PDDF identification experiment but was controlled with its absolute position measured. The SS actuator stage was also controlled with its absolute position, with PDD feedforward compensation activated. Impact excitation using a rubber hammer was applied to the LS actuator stage in the Y direction, while the SS controller forces *F_V_cont_* were recorded corresponding to the relative displacement *u* in the Y direction. The one sampling period delay between the recorded *F_V_cont_* and *u* is compensated for in an off-line data storage process. Figure 10a shows the recorded results in the time domain when the relative position is set as [0, 0, 0], and Figure 10b shows the corresponding Bode diagram of the transfer of $F_{V\_cont}/u$ in the frequency domain. 

Figure 10a shows a clear relationship between error of the LS to SS and the error of the SS. Since the PDDFs have be compensated for, the relationship is caused by VDDFs. 

**Figure 11:**
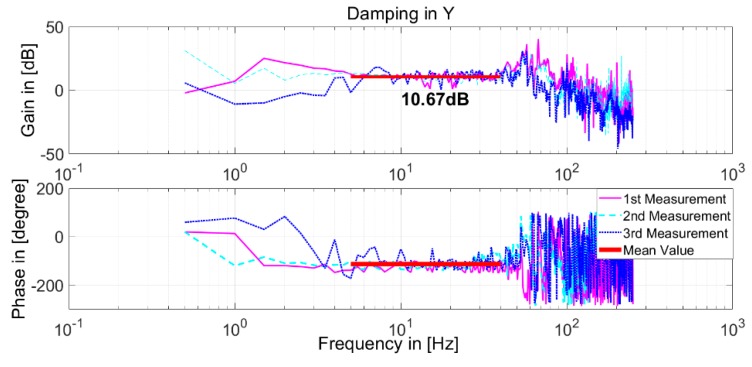
Bode diagram of damping at the relative position [0, 0, 0].

This obtained transfer function $F_{V\_cont}/u$ shown in Figure 10b is multiplied with part A in Equation (3) and integrated. Then, the Bode diagram of Equation (3) can be plotted, as shown in Figure 11.

Figure 11 shows that the gain between 10 Hz and 40 Hz fluctuates close to 10 dB in a narrow range. This means that the damping value *D* at the relative position [0, 0, 0] is approximately constant, as 20log*D* = 10. Thus, by repeating this experiment at 27 relative positions between the LS and the SS actuator stages, the distribution of damping values *D* within the limited 3D motion space of the SS actuator stage could be obtained, specifically, X = (−0.9, 0, 0.9) mm, Y = (−0.9, 0, 0.9) mm, and Z = (−0.4, 0, 0.4) mm. At each relative position, experiments were conducted three times, and the mean value was calculated.

**Figure 12:**
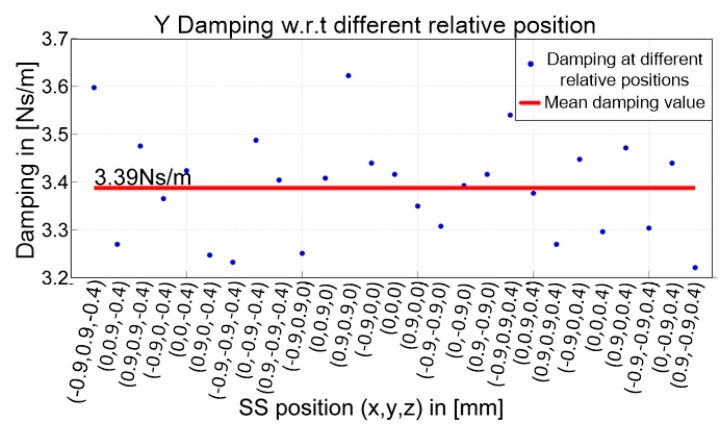
All damping values at different relative positions and their mean values.

All the obtained damping values are plotted in Figure 12. A maximum relative error of 6.7% and standard deviation of 0.33 Ns/m were calculated, which means that damping values at different relative positions are very close to each other. Additionally, it can be concluded that the damping in the Y direction within the 3D space is approximately uniform, given the mean value of 3.39 Ns/m. Thus, the VDDFs are indirectly identified in the form of damping values.

### 4.2. Compensation of Disturbance Forces

#### 4.2.1. Force Feedforward Compensation

**Figure 13:**
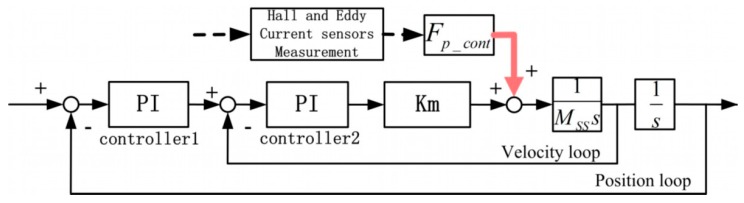
Force feedforward to compensate PDDFs.

A force feedforward table filled with the obtained distribution of PDDFs is applied to compensate PDDFs, indexed with the relative position, as shown in Figure 13. As this feedforward is not part of the closed loop, it will not influence the stability of the SS system.

**Figure 14:**
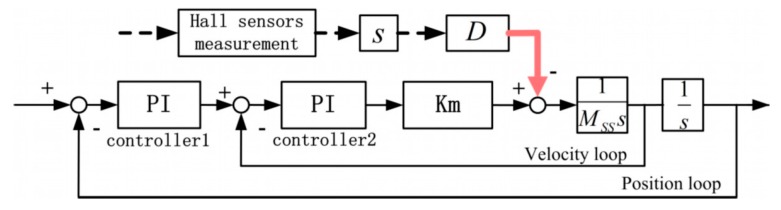
Force feedforward to compensate VDDFs.

Similar to the PDDFs, a feedforward method was applied to compensate the VDDFs, as indexed with the relative velocity in the Y direction between the LS and the SS actuator stages, as shown in Figure 14. 

#### 4.2.2. Verifications

**Figure 15:**
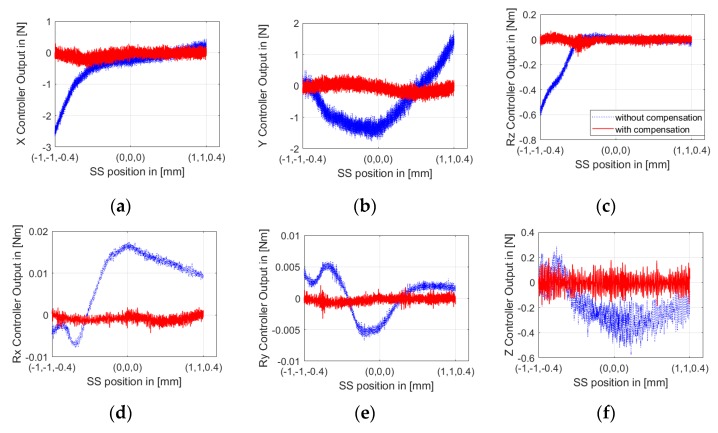
SS controller forces in (**a**) X, (**b**) Y, (**c**) Z, (**d**) Rx, (**e**) Ry, and (**f**) Rz DoF with and without PDDF compensation.

To investigate the effectiveness of the feedforward compensation of PDDFs, the SS actuator stage was moved along the straight line from the relative position of [−1, −1, −0.4] to [1, 1, 0.4] mm, at the same constant velocity of 0.2 mm/s and with PDD compensation activated. The controller forces in 6-DoFs were recorded corresponding to the position of the SS actuator stage relative to the LS actuator stage, as plotted in Figure 15. It is obvious that most PDDFs in 6-DoFs were compensated effectively.

**Figure 16:**
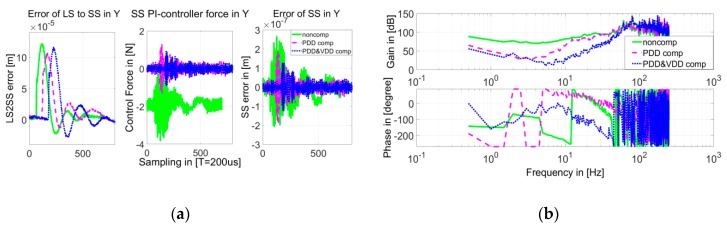
(**a**) Time response and (**b**) frequency response with impact excitation on the Long-Stroke (LS) actuator stage.

To evaluate the effectiveness of the compensations of both PDDFs and VDDFs, the SS actuator stage was positioned at any one position relative to the LS actuator stage, such as [−0.5, −0.5, −0.3] mm. Additionally, impact excitation using a rubber hammer was applied to the LS actuator stage in the Y direction in three conditions: without compensation, with PDD compensation, and with both PDD and VDD compensation. In the meantime, time domain responses were measured, including relative displacement, SS controller forces, and SS actuator stage positioning error, and were plotted as in Figure 16a. The Bode diagram of the frequency transfer function of SS controller force to relative displacement is plotted in Figure 16b.

Though the excitations on the LS actuator stage are not exactly the same in all three conditions, the relative difference between them is smaller than 8%, which almost does not influence the evaluation. In the time domain, the maximum positioning error of the SS actuator stage caused by the impact excitation via the interaction between the two stages becomes better from 260 nm without compensation to 200 nm with PDD compensation, and to 130 nm with further VDD compensation. Additionally, the settling time is shortened significantly. In the frequency domain, when PDD compensation is activated, the gain of interaction transfer is decreased in low frequency range of up to 10 Hz. When VDD compensation is also implemented, the gain is decreased further in the range of up to 45 Hz.

## 5. Conclusions

This paper initially analyzed the mechanism of the interaction in the non-contact DSA systems, based on which the origins of the interaction are clearly classified as the position-dependent and the velocity-dependent disturbance forces, for the first time. Then, the position-dependent disturbance forces were directly identified in the time domain and the velocity-dependent disturbance forces were identified in the form of damping values in the frequency domain. Force feedforward methods were subsequently applied to compensate for both of the disturbance forces. The experimental results demonstrate that the positioning error of the SS caused by such interaction is obviously reduced and the gain of interaction transfer in frequency is greatly decreased. With the aid of the proposed force feedforward method, we can completely compensate for the influence of the weak dynamic interaction between the LS and the SS actuator stages. Therefore, the positioning accuracy of the non-contact DSA systems can be greatly improved without increasing the hardware cost of the system. It could be expected that this research could provide a novel method to further improve nanometer-scale positioning accuracy, such as for lithography. However, when the LS actuator stage moves in a wide range, the external weak disturbance force will directly act on the SS actuator stage without passing through the LS actuator stage. It is then necessary to study such disturbance force compensation methods to further improve the positioning accuracy of non-contact DSA systems.

## Figures and Tables

**Table 1:** Structural and performance parameters of the Dual-Stage Actuator (DSA) system.

	Short Stroke Stage	Long Stroke Stage
Mass	12.11 kg	95.3 kg
Dominant eigenfrequency	Z: 311 Hz; Y: > 1000 Hz	Y: 278 Hz
Required acceleration	X: 60 m/s^2^	60 m/s^2^
Required scan speed	Y: 1 m/s	1 m/s
Required range	X: 2 mm; Y: 2 mm; Z: 0.8 mm	280 mm
Current accuracy	X:80 nm; Y: 60 nm; Z:80 nm;	11 μm
Current bandwidth	X: 50 Hz; Y: 75 Hz; Z:50 Hz	20 Hz
